# Optimization of Short RNA Aptamers for TNBC Cell Targeting

**DOI:** 10.3390/ijms23073511

**Published:** 2022-03-23

**Authors:** Simona Camorani, Annachiara d’Argenio, Lisa Agnello, Roberto Nilo, Antonella Zannetti, Luis Exequiel Ibarra, Monica Fedele, Laura Cerchia

**Affiliations:** 1 National Research Council (CNR), Institute of Experimental Endocrinology and Oncology “Gaetano Salvatore” (IEOS), 80131 Naples, Italy; a.dargenio@ieos.cnr.it (A.d.); lisa.agnello@ieos.cnr.it (L.A.); r.nilo@studenti.unina.it (R.N.); mfedele@unina.it (M.F.); 2 Department of Precision Medicine, University of Campania “L. Vanvitelli”, 80138 Naples, Italy; 3 National Research Council (CNR), Institute of Biostructures and Bioimaging (IBB), 80145 Naples, Italy; antonella.zannetti@ibb.cnr.it; 4 Institute of Environmental Biotechnology and Health (INBIAS), National University of Rio Cuarto (UNRC), National Council for Scientific and Technological Research (CONICET), Río Cuarto X5800BIA, Argentina; libarra@exa.unrc.edu.ar

**Keywords:** RNA aptamer, active targeting, aptamer structures, TNBC

## Abstract

Triple-negative breast cancer (TNBC) is an aggressive cancer with limited targeted therapies. RNA aptamers, suitably chemically modified, work for therapeutic purposes in the same way as antibodies. We recently generated 2′Fluoro-pyrimidines RNA-aptamers that act as effective recognition elements for functional surface signatures of TNBC cells. Here, we optimized three of them by shortening and proved the truncated aptamers as optimal candidates to enable active targeting to TNBC. By using prediction of secondary structure to guide truncation, we identified structural regions that account for the binding motifs of the full-length aptamers. Their chemical synthesis led to short aptamers with superb nuclease resistance, which specifically bind to TNBC target cells and rapidly internalize into acidic compartments. They interfere with the growth of TNBC cells as mammospheres, thus confirming their potential as anti-tumor agents. We propose sTN145, sTN58 and sTN29 aptamers as valuable tools for selective TNBC targeting and promising candidates for effective treatments, including therapeutic agents and targeted delivery nanovectors.

## 1. Introduction

Triple-negative breast cancer (TNBC) is characterized by the absence of estrogen receptor (ER), progesterone receptor (PR) and human epidermal growth factor receptor 2 (HER2), making hormone therapy and HER2-targeting drugs not useful for treatment [1,2]. Due to the lack of well-defined TNBC biomarkers, which may lead to targeted therapy, chemotherapy still remains the main treatment option for early-stage and advanced tumors, despite its efficacy being limited by elevated toxicity in normal cells, poor bioavailability and drug resistance [3,4,5].

In recent years, the cell-SELEX (Systematic Evolution of Ligands by Exponential Enrichment) technology has been widely used for the discovery of biomarkers. Indeed, it allows the selection of oligonucleotide aptamers against a specific cancer-cell type and uses them to identify novel protein biomarkers of the tumor cell membrane, whose functions in tumor development may also be unknown [6]. DNA or RNA aptamers, based on their sequence, fold into unique three-dimensional structures, which recognize their targets with high affinity and specificity in a manner similar to antibody-antigen interactions [7]. Cell-targeting aptamers are emerging as a new class of efficacious recognition molecules that find application in different targeted cancer therapy strategies. These include the use of aptamers as stand-alone antagonistic agents, which interfere with the activity of the receptor target, or as intracellular delivery vehicles of therapeutic payloads, which are directly conjugated to aptamers or encapsulated into aptamer-equipped nanovectors [6,8]. Notably, aptamer-functionalized nanotherapeutics exhibit the potential to overcome current chemotherapeutic limitations because they enable the controlled and efficacious release of the loaded drug to the tumor, thus significantly improving its bioavailability and therapeutic efficacy while reducing adverse side effects [9]. Our group has recently shown the ability of a nuclease-resistant RNA aptamer targeting the epidermal growth factor receptor (EGFR) to deliver cisplatin-loaded polymeric nanoparticles specifically to EGFR-positive TNBC implanted in mice, thus causing the safe accumulation of the drug selectively at the tumor site [10]. To increase the currently available repertoire of agents for TNBC-specific targeting, using a human TNBC cell line as a target for SELEX, we have recently generated a panel of six 2′Fluoro-pyrimidines (2′F-Pys) RNA aptamers with unequivocal efficacy in targeting human cell lines and tissues covering different TNBC subtypes and discriminating them from both non-tumor samples and triple-positive breast cancers (TPBC, ER+, PR+, HER2 over-expression) [11]. These aptamers, binding to cytomembrane proteins, represent very promising tools for molecular recognition in novel aptamer-targeted nanotherapy for TNBC. In order to enable the efficient functionalization of the nanoparticle surface with these molecules, here we attempted the generation of shortened versions of 3 of the 6 aptamers that preserve the cell-targeting properties of the full-length (84 mer) moieties generated by the SELEX process. Undoubtedly, minimized sequences have several advantages over the original longer sequences. First, they result in easier chemical synthesis with higher yield and lower costs; the presence of modified nucleotides in the entire aptamer sequence, as, for instance, 2′F-Pys, further increases the manufacturing expense. Furthermore, from a functional point of view, small-size aptamers show reduced systemic toxic effects, higher tissue penetration and easier conjugation to secondary therapeutics [12,13,14,15]. A full-length aptamer sequence is comprised of highly structured regions, which are more likely involved in target binding, and non-essential regions, generally poorly structured, which could interfere with the target recognition. Thus, removing unnecessary nucleotides may result in a lower steric hindrance and fewer unproductive intramolecular interactions, allowing the truncated aptamer to bind more readily to the target with a significant increase in binding affinity [16]. 

In this study, in order to identify the potential binding segment within the full-length anti-TNBC TN145, TN58, TN29 aptamers, we compared the secondary structures predicted by three software packages (RNAstructure, Mfold and ViennaRNA package) with the lowest free energy and thus highest stability. Common highly structured motifs among these structures were identified, and the truncated sequences were chemically synthesized. The shorter sTN145, sTN58, sTN29 aptamers, consisting of 49, 45 and 41 nucleotides (nt), respectively, were experimentally verified for their cell binding and internalization, nuclease resistance and ability to inhibit mammosphere-forming properties of TNBC cell lines. Importantly, they preserve efficacious targeting and rapid cell uptake, as well as anti-tumor activity as the parental moieties. In addition, because the 2′F-Py modifications, they show an exquisite resistance in human serum.. Overall, they represent optimal candidates to enable active targeting to TNBC.

## 2. Results 

### 2.1. Identification of Potential Binding Sites

In order to optimize long aptamer sequences for biomedical applications, secondary structure information can be used to truncate conserved structured stem-loop regions that are likely involved in target recognition to: (1) eliminate sequences that are useless and may even reduce affinity and (2) allow chemical synthesis at low cost and in large-scale quantities [17]. We previously selected TN145, TN58 and TN29 aptamers (84 nt in length) specifically targeting TNBC cells, which consist of a 40-mer variable central region flanked by 2 fixed sequences of 21 and 23 nt at the 5′ and 3′ terminal ends, respectively, working as primer binding sites for PCR (Supplementary Table S1) [11]. First, secondary structures of full-length aptamers, theoretically predicted by computational approach, were used to identify potential binding motifs and rationally truncate original aptamers. For each sequence, the secondary structure was predicted by ViennaRNA Package, RNAstructure and Mfold, three frequently used simulation software based on the minimum free energy (MFE) method, the most popular structure prediction algorithm for sequence folding. MFE-based approaches analyze all possible folding conformations for a given sequence and determine structures with MFE, an indicator of structural stability [18]. 

**Figure 1:**
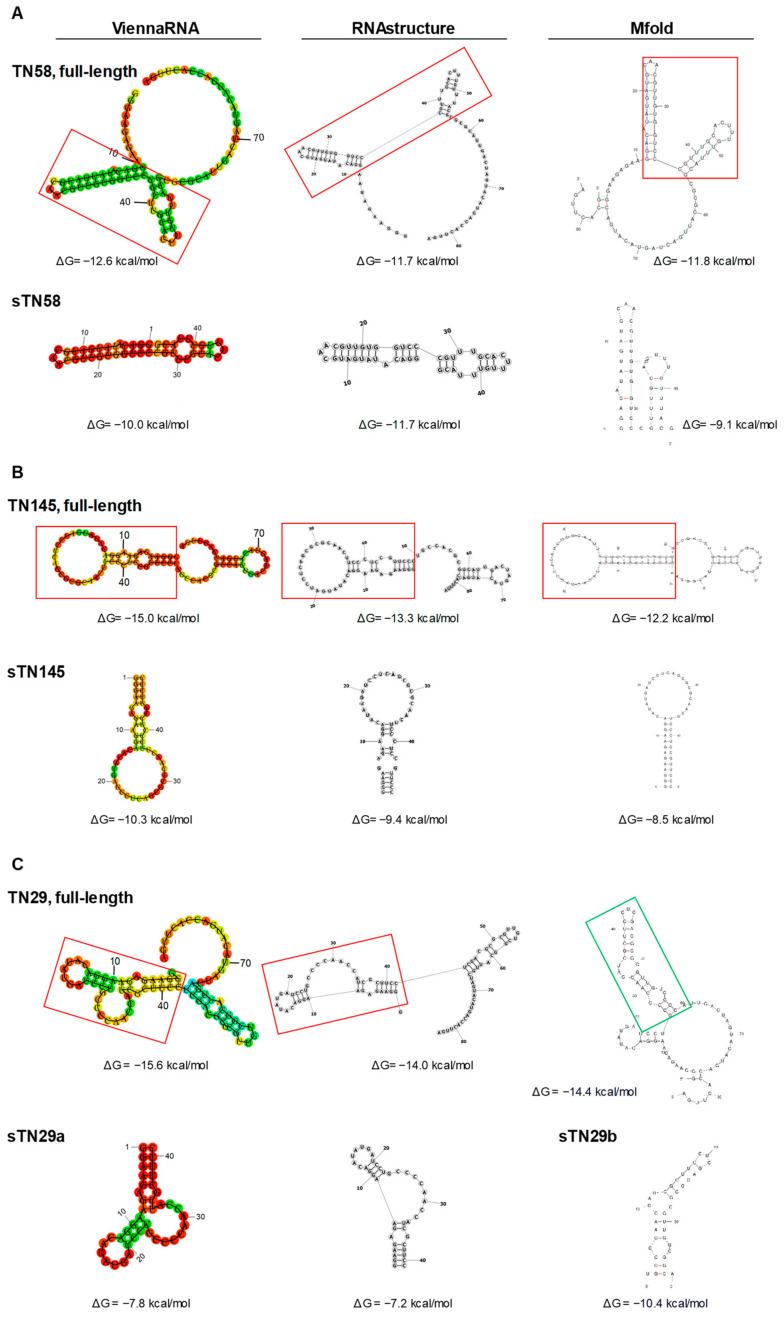
Secondary structure of full-length and short aptamers. Secondary structures of TN58 (**A**), TN145 (**B**) and TN29 (**C**) aptamers were predicted by ViennaRNA Package, RNAstructure and Mfold. ΔG values are reported. Boxes indicate the potential binding sites.

Upon loading the full-length TN145, TN58 and TN29 aptamer sequences (Supplementary Table S1) in the three programs, we considered the most stable structure, among the various possible folds for each sequence, characterized by the lowest free energy change (ΔG). By comparing the secondary structures assigned by the three programs (Figure 1), we individuated the common structured regions and checked that they retained their folding even if the rest of the nucleotides were eliminated. 

Regarding the TN58 aptamer, the most stable structure (ΔG values of approximately −12 Kcal/mol), predicted by each of the three software, was characterized by two adjacent stem-loops, spanning nt 11 to nt 55, and an unstructured region contributed by the remaining nucleotides at 5′ and 3′ ends (Figure 1A, upper). The structured region, corresponding to a shorter aptamer (named sTN58), as predicted by ViennaRNA Package, RNAstructure and Mfold, is shown in Figure 1A (lower). 

Similarly, in the case of the TN145 aptamer, the software predicted a common secondary structure that contained 2 structured regions at the 5′ and 3′ extremities (Figure 1B, upper). The biggest stem-loop region (49 nt) at the 5′ extremity was chosen as a potential binding site because, as opposed to the hairpin loop at the 3′ extremity, which is almost entirely contributed by the primer sequence, the 5’ extremity is contributed by the 5′ primer sequence (21 nt) and a large part of the central random region (28 nt). Furthermore, it is endowed with a lower ΔG than the region at the 3′ extremity. Thus, the aptamer sequence from 1 to 49 nt (named sTN145) was used for the following validation tests of targeting efficiency (Figure 1B, lower).

Compared to what was observed for TN58 and TN145, the structure at the lowest ΔG predicted for TN29 by ViennaRNA Package and RNAstructure was different from that predicted by Mfold (Figure 1C, upper), likely reflecting an alternative implementation of the same model for each program [19,20]. The lowest ΔG structure common in ViennaRNA Package and RNAstructure (ΔG values of approximately −15 Kcal/mol) was characterized by 2 continuous structured stem-loops (spanning nt 2 to nt 65) and an unstructured region at the 3′ end. The first stem-loop, at the 5′ end of the sequence (from nt 2 to nt 42), includes an internal loop and a bulge to which the 5′ primer sequence and 21 nucleotides of the variable region contribute. The second stem-loop (from nt 43 to nt 65) consists almost entirely of the random region (Figure 1C, upper). Conversely, the Mfold software predicted a secondary structure (ΔG values of −14.4 Kcal/mol) consisting of a central loop with a longer (from nt 25 to nt 60) and a shorter (from nt 10 to nt 24) branch (Figure 1C, upper). For efficacy testing, we started with the first stem-loop (from nt 2 to nt 42) in the structured region at the 5′ extremity as predicted by ViennaRNA Package and RNAstructure (named sTN29a) and the longer branch, consisting of the variable region, of the loop structure predicted by Mfold (named sTN29b) (Figure 1C, lower).

### 2.2. The Short Aptamers Specifically Target TNBC Cells

**Figure 2:**
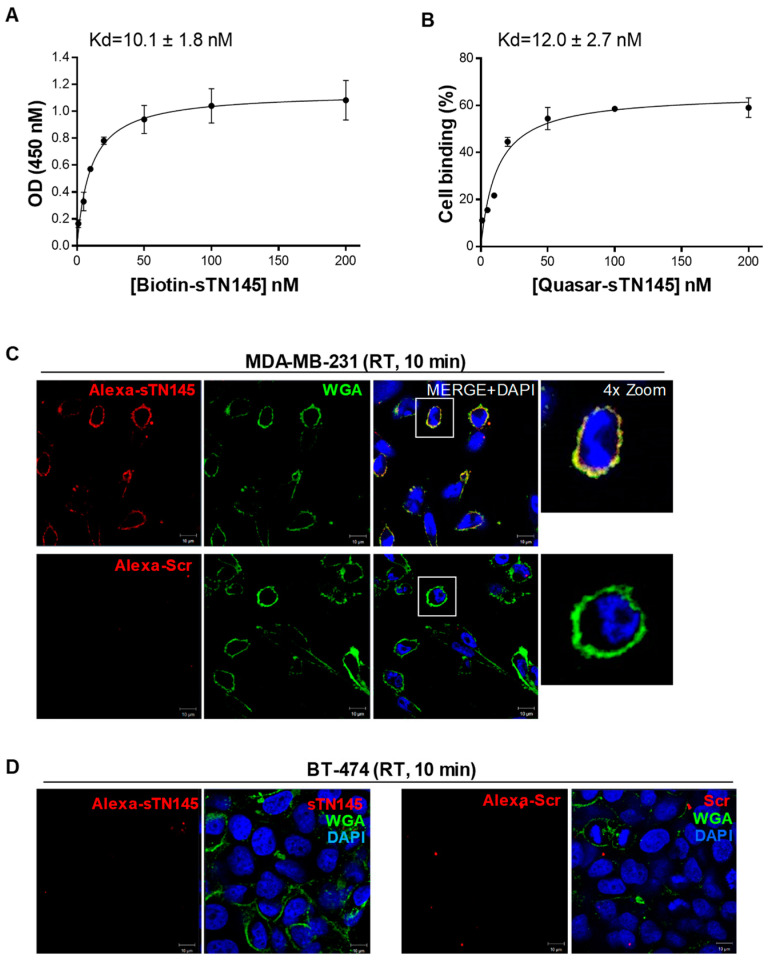
Cell binding of the sTN145 aptamer. Binding curves of sTN145, 5′-biotinylated (**A**) or 5′-Quasar 670-labeled (**B**) on MDA-MB-231 cells for calculation of the apparent Kd of aptamer-cell interaction. Binding was analyzed using streptavidin-biotin-based (**A**) or flow cytometric (**B**) assays. Data shown are mean ± SD of three independent experiments. (**C**,**D**) Representative confocal images of MDA-MB-231 (**C**) and BT-474 (**D**) cells incubated for 10 min at RT with Alexa 647-labeled sTN145 or Scr. After washing and fixation, cells were labeled with WGA-488 (green) to visualize the cell membrane and with DAPI (blue) to stain nuclei. Alexa 647-labeled aptamers are displayed in red. All digital images were captured at the same setting to allow direct comparison of staining patterns. Magnification 63×, 1.0× digital zoom, scale bar = 10 μm. At least three independent experiments were performed. In (**C**), co-localization results appear yellow in the merged images. Inset: 4× digital zoom.

The truncated sequences individuated as potential binding sites were chemically synthesized and tested to verify whether they preserve the binding affinity of the correspondent parental sequences to human TNBC cells or, eventually, bind them even better. To this aim, we used MDA-MB-231 cells, which represented a well-established model of aggressive TNBC [21,22,23] and were previously used as a target in cell-SELEX that led to the identification of the long version of these aptamers [11]. Given the complexity of the target, different types of assays were harnessed to assess the targeting efficiency of the short aptamers both in terms of affinity and specificity. Regarding sTN145, we used a modified version on the 5′-end with biotin and fluorescent Quasar 670 in streptavidin-biotin-based (colorimetric) and flow cytometric assays, respectively, to evaluate its binding affinity (Kd values). A 5′ biotin- and Quasar-labeled scrambled sequence (Scr), which contains 2′F-Pys all along the sequence and is similar in length to the short sequences to be tested, was used as a negative control. As shown, sTN145 aptamer was bound to MDA-MB-231 cells with high affinity and Kd values of 10.1 ± 1.8 nM and 12.0 ± 2.7 nM, as assessed by colorimetric (Figure 2A) and flow cytometric (Figure 2B) assays, respectively. These values were lower than those obtained with the original full-length aptamer (Kd ranging from 26.9 ± 3.7 to 37.4 ± 3.5) [11], thus indicating not only that the essential nucleotides involved in the binding were preserved during the shortening but also that the removal of unnecessary nucleotides led to improved binding affinity. Furthermore, the cell-targeting specificity of sTN145 was confirmed by confocal microscopy using TNBC and non-TNBC cell lines. To this aim, we internally labeled the aptamer with Alexa 647, as we previously settled in our laboratory [11] and verified that the internal introduction of fluorescent dyes did not affect the folding of the aptamer and consequently the recognition of the target cells (Supplementary Figure S1). Then, MDA-MB-231 cells were incubated with Alexa 647-sTN145 for 10 min at room temperature (RT) and stained with wheat germ agglutinin (WGA)-Alexa Fluor 488 to allow for the identification of the cell surface. As shown in Figure 2C, the sTN145 aptamer is clearly localized on the cell surface, thus in agreement with the ability of the long TN145 to bind to a not-yet-known protein expressed on the surface of TNBC cells [11]. As expected, very little or no signal was observed with Scr (Figure 2C). No binding of sTN145 was observed onto BT-474 cells (ER+, PR+, HER2 overexpression) (Figure 2D), which had previously been used in the counterselection steps [11], thus demonstrating the efficacy of the short aptamer, as the long one, to discriminate TNBC from TPBC cells.

**Figure 3:**
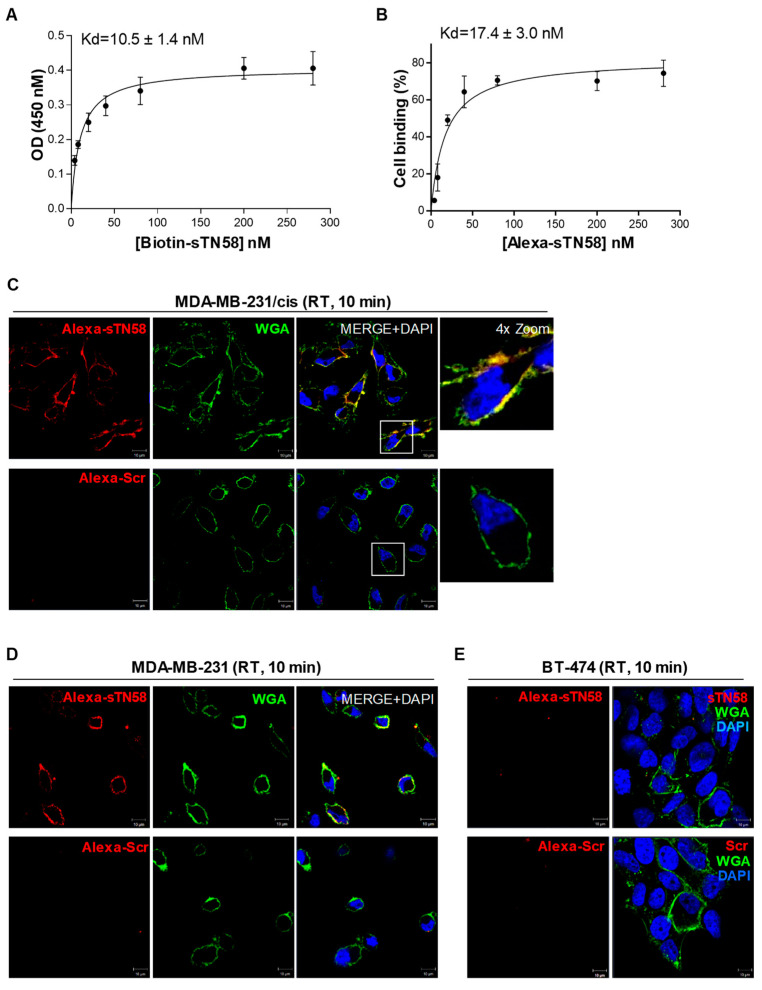
Cell binding of sTN58 aptamer. Binding curves of sTN58, 5′-biotinylated (**A**) or Alexa 647-labeled (**B**) on MDA-MB-231/cis cells for the calculation of the apparent Kd of aptamer-cell interaction. Binding was analyzed using streptavidin-biotin-based (**A**) or flow cytometric (**B**) assays. Data shown are mean ± SD of four independent experiments. (**C**–**E**) Representative confocal images of MDA-MB-231/cis (**C**), MDA-MB-231 (**D**) and BT-474 (**E**) cells incubated for 10 min at RT with Alexa 647-labeled sTN58 or Scr. Alexa 647-labeled aptamer, WGA-488, and nuclei are visualized in red, green, and blue, respectively. All digital images were captured at the same setting to allow direct comparison of staining patterns. Magnification 63×, 1.0× digital zoom, scale bar = 10 μm. At least three independent experiments were performed. In (**C**,**D**), co-localization results appear yellow in the merged images. Inset: 4× digital zoom.

We have previously generated a cisplatin-resistant MDA-MB-231 (MDA-MB-231/cis) cell line and, notably, showed that the full-length TN58 aptamer displayed a higher affinity for chemo-resistant cells than parental MDA-MB-231 cells [11]. Therefore, in order to verify the targeting efficiency of the sTN58 aptamer, we determined Kd values on chemo-resistant cells by colorimetric (Figure 3A) and flow cytometric (Figure 3B and Supplementary Figure S2) assays. Because comparable results were obtained by the flow cytometric binding assays performed with the Quasar 670- and Alexa 647-labeled sTN145, as shown above, we used Alexa 647-sTN58 for further characterization requiring a fluorescent version of this aptamer. As shown, aptamer sTN58 bound efficiently to MDA-MB-231/cis with Kd values of 10.5 ± 1.4 nM (colorimetric assay, Figure 3A) and 17.4 ± 3.0 nM (flow cytometry, Figure 3B and Supplementary Figure S2), which was somewhat comparable to that observed by the full-length TN58 aptamer on MDA-MB-231/cis (9.3 ± 1.8 nM, flow cytometry) [11]. Furthermore, sTN58, as the long sequence, displayed a higher binding affinity on chemo-resistant cells than parental MDA-MB-231, likely reflecting enrichment of the aptamer target on the surface of the MDA-MB-231/cis cells (Supplementary Figure S3). Confocal microscopy analyses indicated the ability of Alexa-labeled sTN58 to decorate the surface of both chemo-resistant (Figure 3C) and parental MDA-MB-231 cells (Figure 3D) but not BT-474 cells (Figure 3E). No binding to the above cell lines was observed by the Scr negative control (Figure 3C–E). These results indicate sTN58 as a valid tool for future applications. 

**Figure 4:**
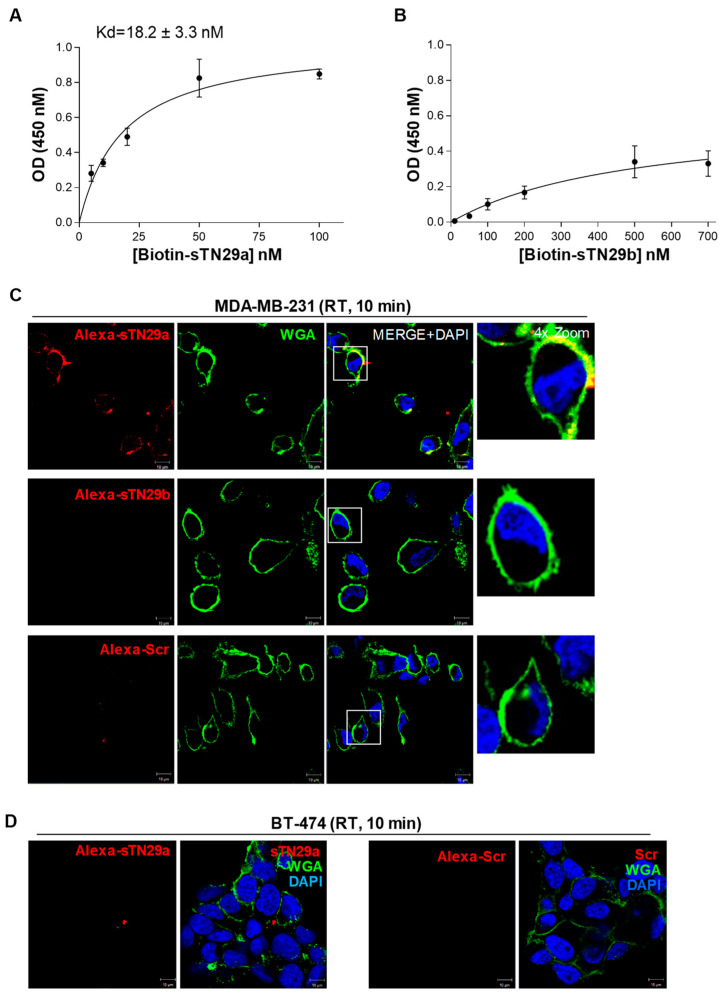
Cell binding of the sTN29 aptamer. Binding curves of the 5′-biotinylated sTN29a (**A**) or sTN29b (**B**) on MDA-MB-231 cells for calculation of the apparent Kd of aptamer-cell interaction. Binding was analyzed using the streptavidin-biotin-based assay. Data shown are mean ± SD of three independent experiments. (**C**,**D**) Representative confocal images of MDA-MB-231 (**C**) and BT-474 (**D**) cells were incubated for 10 min at RT with the indicated Alexa 647-labeled aptamers. Alexa 647-labeled aptamer, WGA-488, and nuclei are visualized in red, green, and blue, respectively. All digital images were captured at the same setting to allow direct comparison of staining patterns. Magnification 63×, 1.0× digital zoom, scale bar = 10 μm. At least three independent experiments were performed. In (**C**), co-localization results appear yellow in the merged images. Inset: 4× digital zoom.

With the aim of ascertaining the binding properties of the two truncated versions of TN29, sTN29a and sTN29b (see predicted structures in Figure 1), MDA-MB-231 cells were incubated with 5′-biotinylated aptamers and analyzed by streptavidin-biotin-based assays. As shown, sTN29a aptamer bound to MDA-MB-231 cells at high affinity, with a Kd value of 18.2 ± 3.3 nM (Figure 4A) that was comparable to the long sequence [11], whereas a Kd value above 500 nM was obtained with sTN29b (Figure 4B). Consistently, fluorescent-labeled sTN29a, but not sTN29b, bound to MDA-MB-231 cells (Figure 4C and Supplementary Figure S4), discriminating them from BT-474 cells (Figure 4D). These results indicate that sTN29a (hereafter referred to as sTN29) preserves the efficient cell targeting of the long sequence and thus was chosen for further characterization. We cannot exclude that the yet-unexplored second stem-loop, from nt 43 to nt 65, of the TN29 aptamer (see Figure 1C, ViennaRNA and RNA structure) could in some way participate in cell recognition.

Furthermore, we verified that the short sTN145 and sTN29 aptamers were capable of targeting MDA-MB-231/cis cells as the full-length counterparts. As shown (Supplementary Figure S3), binding curves obtained by colorimetric assays revealed that the two short aptamers bind to chemo-resistant cells with an affinity comparable to that displayed on parental cells, thus reflecting the targeting specificity of the long aptamers.

Moreover, the short sTN145, sTN29 and sTN58 aptamers were still able to bind to TNBC BT-549 cells (Supplementary Figure S5) as the long moieties [11]. These cells share the same mesenchymal and aggressive phenotype of MDA-MB-231 cells [22,23].

Collectively, these results indicate that computational secondary structure analysis-driven truncation is an effective strategy for identifying short binding motifs. Indeed, sTN145, sTN58 and sTN29 preserve and sometimes improve the binding affinity and specificity of the long parental moieties with the undoubted advantage of a lower size for better applicability. 

### 2.3. The Short Aptamers Actively Internalize into TNBC Cells

**Figure 5:**
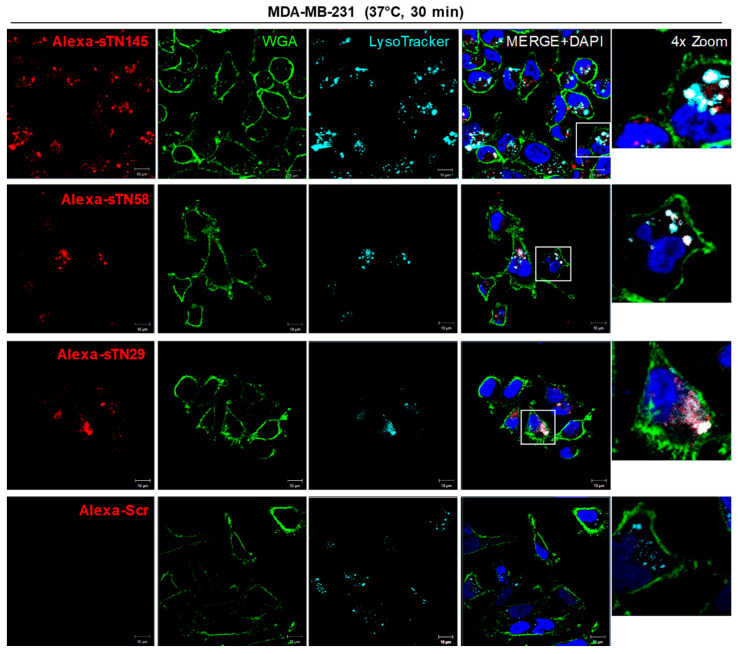
Cell uptake of sTN145, sTN58 and sTN29 aptamers. Representative confocal images of MDA-MB-231 cells incubated for 30 min at 37 °C with Alexa 647-labeled sTN145, sTN58, sTN29 or Scr after treatment with LysoTracker to detect acidic organelles inside the cells. Alexa 647-labeled aptamer, WGA-488, LysoTracker, and nuclei are visualized in red, green, light blue, and blue, respectively. Co-localization results appear white in the merged images. Magnification 63×, 1.0× digital zoom, scale bar = 10 μm. Inset: 4× digital zoom. At least three independent experiments were performed.

A subset of cell surface-targeting aptamers is known to actively enter the target cell, usually via receptor-mediated endocytosis. This makes them useful for delivering therapeutic payloads specifically into target cancer cells following their direct conjugation to the drug or different drug-loaded nano-formulations [6,24]. In order to utilize the new truncated sequences for functionalizing drug-loaded nanoparticles and driving them to TNBC cells, we evaluated their uptake into MDA-MB-231 cells by confocal microscopy imaging. To this aim, cells pre-treated with LysoTracker to stain late endosomes and lysosomes compartments were incubated with Alexa 647-labeled sTN145, sTN58 or sTN29 aptamers for 30 min at 37 °C. As shown, all 3 aptamers, colocalizing with cell surface WGA marker at 10 min incubation (Figure 2, Figure 3 and Figure 4), internalize within the target cells by prolonging the incubation, and accumulate in compartments positive for LysoTracker (Figure 5). As expected, no signal was observed in the presence of the Alexa 647-Scr control under the same experimental conditions (Figure 5). The cellular targeting and internalization abilities of these short aptamers make them potentially useful ligands for TNBC drug delivery.

### 2.4. The Short Aptamers Are Highly Resistant to Nuclease Degradation

The stability of RNA aptamers in human biological fluids represents a fundamental requirement for successful in vivo applications. Their serum stability is determined by backbone composition and limited by nuclease cleavage. Indeed, unmodified RNA aptamers are highly susceptible to nuclease degradation in human serum, with in vitro half-lives of a few seconds. Conversely, chemically modified RNAs commonly bearing substitution at the 2′-position of the ribose unit with fluoro, methoxy, thio or amino groups have high resistance to nucleases, with in vitro half-lives of several hours [25,26]. The full-length TNBC aptamers have been selected starting from a library of 2′F-Py RNAs by using a mutant of the T7 RNA polymerase that incorporates modified nucleotides during the in vitro transcription [11].

**Figure 6:**
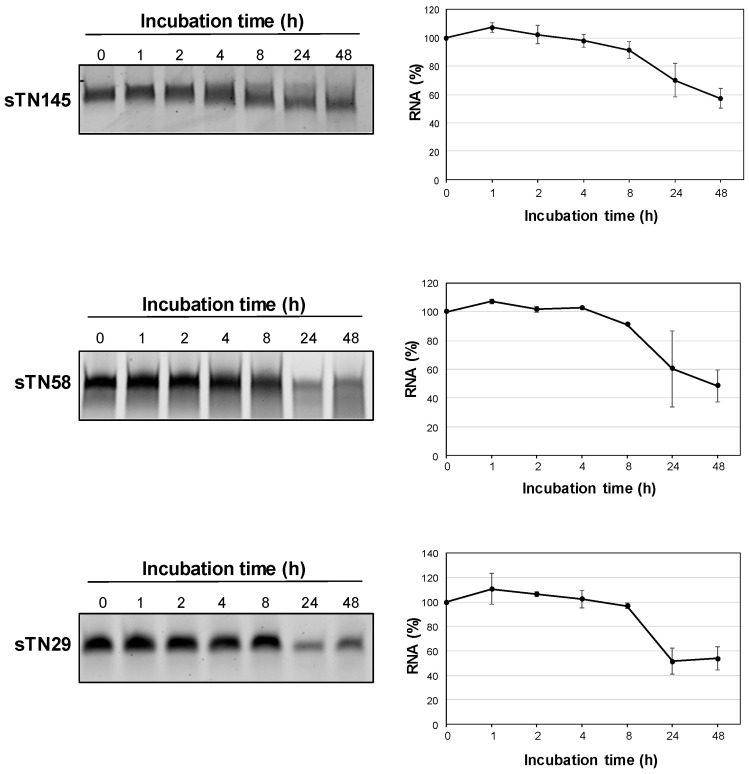
In vitro serum stability analysis of sTN145, sTN58 and sTN29 aptamers. (**Left**) Denaturing PAGE of the short aptamers following incubation with 80% human serum at the indicated times. Depicted results represent one of three typical experiments performed. The 3 short aptamers contain 2′F-Pys in the entire sequence to increase nuclease resistance. (**Right**) Band intensity was quantified using ImageJ (v1.46r) at each time point and expressed as a percentage with respect to time 0 RNA. Note that time 0 was taken after 1 h incubation with proteinase K. Data shown are mean ± SD of three independent experiments.

However, in order to test whether the short sTN145, sTN58, and sTN29 aptamers are adequately resistant to nuclease degradation, they were incubated at 37 °C in 80% human serum for increasing times, up to 48 h, and the integrity of RNA samples was analyzed on denaturing polyacrylamide gel electrophoresis (PAGE). As shown in Figure 6, the 3 shortened aptamers remained almost completely stable up to 8 h and then slowly degraded, with approximately 50% of undegraded, intact RNA still detectable at 48 h. This indicates that the 3 short aptamers, accordingly to their content in 2′F-Pys (Supplementary Table S1), show an exquisite stability in human serum, thus allowing further development for clinical use. 

### 2.5. The Short Aptamers Efficiently Target TNBC CS.C.s

We have previously shown that our TNBC-targeted full-length aptamers were able to affect the malignancy of cancer cells. In particular, they were able to inhibit the ability of TNBC cells to form mammospheres in vitro [11]. The mammosphere formation assay (MFA) is a useful method for studying cancer stem cells (CSCs) because the cells that initiate the sphere are surrogates for CSCs in culture [27]. Therefore, we have shown that these aptamers target TNBC CSCs, which are associated with aggressive and metastatic features of cancer [11].

**Figure 7:**
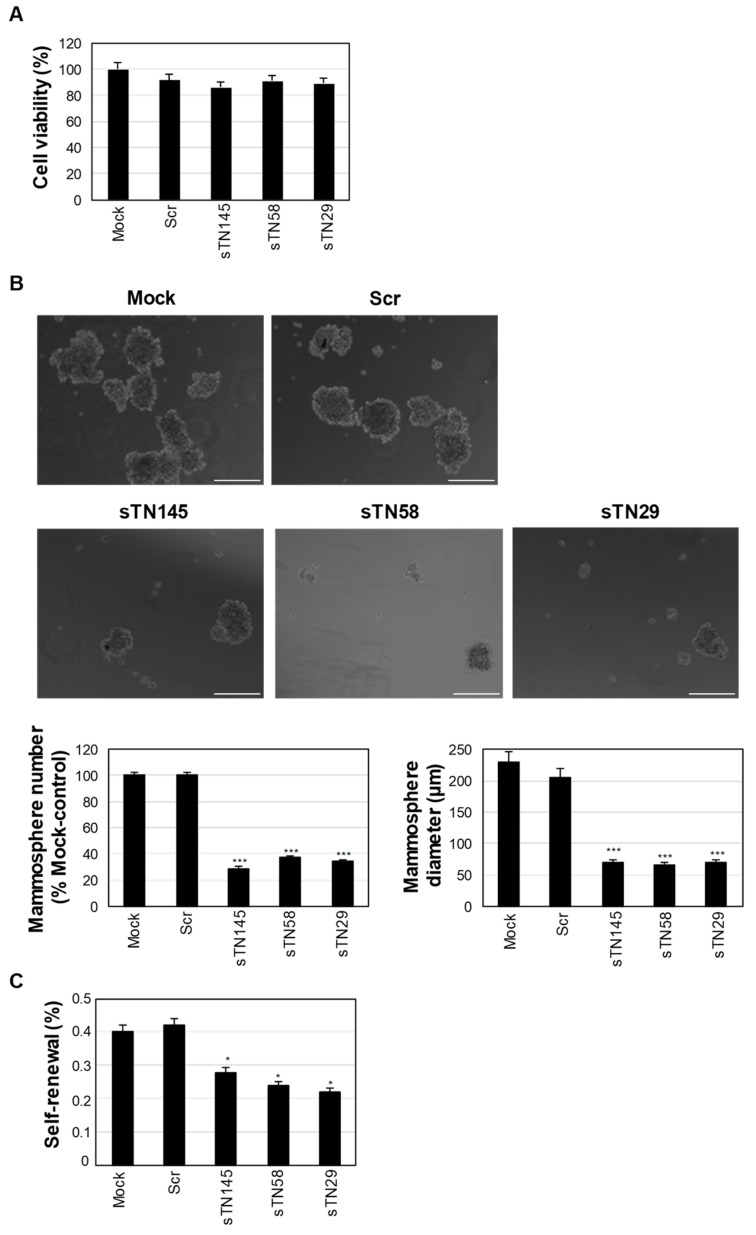
Effect of sTN145, sTN58 and sTN29 aptamers on mammosphere-forming efficiency of MDA-MB-231 cells. (**A**) Viability of MDA-MB-231 cells mock-treated or treated with the indicated aptamers for 24 h. Data are expressed as a percentage of viable treated cells with respect to mock-treated cells. (**B**) Representative phase-contrast images of MDA-MB-231 cells, previously treated for 24 h with sTN145, sTN58, sTN29 or Scr, after 10 days of growth in stem-permissive conditions. Magnification 10×, scale bar = 200 μm. Cell treatment with the short TNBC aptamers, but not the Scr, inhibits both the number, expressed as a percentage with respect to mock-control cells (left) and diameter (right) of mammospheres. (**C**) Mammosphere self-renewal capacity is expressed as a number of secondary mammosphere population (P1)/cell-seeded × 100. (**A**–**C**) Bars depict mean ± SD of three independent experiments. In (**B**,**C**), *** *p* < 0.001; * *p* < 0.05 relative to mock-control cells. No statistically significant variations between Scr and mock-treatment were obtained.

To investigate whether a transient treatment of each of the short aptamers sTN145, sTN58 and sTN29 was capable and sufficient to inhibit CSCs in TNBC, we incubated MDA-MB-231 cells with them for 24 h, then removed the aptamers and performed MFA. As shown in Figure 7A, this treatment did not affect cell survival with any of the three short aptamers. However, each was able to drastically reduce the number (up to 30%), diameter (about 30%) and self-renewal ability (up to 50%) of mammospheres compared to mock and Scr-treated controls (Figure 7B,C), suggesting that they can directly reduce the CSCs subpopulation, delay their growth and affect their capacity to increase their initial number in subsequent passages in culture.

## 3. Discussion 

TNBC is an aggressive cancer with limited targeted therapies because of the lack of ER, PR and HER2 on its cell surface, which makes hormone therapy and HER2-targeting drugs not useful for treatment. Thus, the identification of novel agents for specific TNBC recognition and treatment is desperately needed. Using a TNBC cell-SELEX approach, we have recently generated a panel of 6 2′F-Pys RNA aptamers (TN2, TN3, TN20, TN29, TN58, TN145) able to bind at high efficiency to surface proteins of human TNBC cells without recognizing non-malignant cells or non-TNBC breast cancer cells representative of luminal A and HER2-positive subtypes [11]. These aptamers represent a highly innovative and useful component for ensuring the TNBC selectivity of nanotherapeutics; nevertheless, their chemical synthesis is hampered by the 84 nt-length. Here, we generated shorter versions of TN145, TN58 and TN29 aptamers than their full-length counterparts, thus more readily chemically synthesized and modifiable, to enable efficient conjugation to drug-loaded nanoparticles for controlled and effective release of their cargo to TNBC. Truncated sequences have several advantages over the original longer sequences, apart from easier chemical synthesis at lower costs, including reduced systemic toxic effects, higher tissue penetration and easier conjugation to secondary therapeutics.

An approach based on the prediction of secondary structure by three software packages (RNAstructure, Mfold and ViennaRNA package) was used to guide the truncation of the previously characterized full-length aptamers. We identified structural regions that account for the binding motifs of the full-length aptamers. The constant regions of aptamer sequences have often been found to participate in the functional secondary structures by interacting with the random region [28,29,30,31]. We also found that in the three short aptamers, the 5′ priming sequence is partly involved in the functional binding site. Their chemical synthesis led to short aptamers, namely sTN145, sTN58 and sTN29, of 49, 45 and 41 nt, respectively, which preserve the cell-binding and discriminating properties of the parental moieties, proving that the binding mechanism of aptamers does not involve the entirety of the sequence. Colorimetric and flow cytometric assays, performed with biotinylated and fluorescent-labeled aptamers, respectively, showed that sTN145, sTN58 and sTN29 specifically bind to TNBC MDA-MB-231 and BT-549 cells, as well as cisplatin-resistant derivatives, with high affinity, displaying Kd values ranging from 10 to 18 nM, which were comparable or even lower (as for sTN145) than those of parental moieties. Notably, we show that the 3 short aptamers, upon binding to cell surface portals, whose identification/validation is currently underway, readily enter MDA-MB-231 target cells, thus showing great potential for cargo delivery. Whether the further systematic truncation of the short aptamers may lead to minimal functional variants remains to be ascertained. Notably, the actual length is compatible with their efficient conjugation to drug-loaded nanosystems. Indeed, recent results from our group show the ability of these aptamers to confer selective TNBC cell targeting to photosensitizers based on conjugated polymer-nanoparticles. Once used to decorate the nanovectors, the short aptamers efficiently deliver the nanoparticles to TNBC cells, promote their cell uptake and ultimately strongly improve the efficacy of photodynamic therapy in chemo-resistant TNBC cell lines [32]. Importantly, confirming the validity of our truncation strategy, we showed that the short aptamers, while binding to the cell surface target protein at high affinity, are also capable of interfering with the ability of TNBC cells to form mammospheres in culture, thus definitely proving that they account for the functional region of the parental long moieties. Furthermore, the presence of 2′F-Pys in the sequence confer high stability to these molecules, as demonstrated by their poor degradation in human serum even after prolonged incubation. 

Given the validity of the proposed approach, the optimization of the other 3 previously generated TNBC aptamers (TN2, TN3 and TN20) by their shortening is currently underway. It will supply multiple tumor-targeting agents with the possibility of using them in different combinations depending on distinct molecular and/or clinical TNBC phenotypes.

The availability of nuclease-resistant short aptamers will certainly enhance their further use both to identify novel TNBC biomarkers and to develop smart aptamer-based conjugates [33,34] and nanoplatforms [10,32] for the treatment of these tumors.

## 4. Materials and Methods

### 4.1. Cell Lines and Culture Conditions

Growth conditions for human TNBC MDA-MB-231 and BT-549 and TPBC BT-474 cells (American Type Culture Collection, Manassas, VA, USA) were previously reported [11]. The MDA-MB-231 cisplatin-resistant cell line (MDA-MB-231/cis) was generated by chronic treatment with cisplatin, as previously described [11].

### 4.2. Aptamer Synthesis, Use Conditions and Secondary Structure Prediction

In this step, 2′F-Py-containing RNAs, either unmodified or conjugated at 5′ extremity with biotin or fluorescent Quasar 670 dye, were synthesized by LGC Biosearch Technologies (Risskov, Denmark, sTN145, sTN58, Scr) or Integrated DNA Technologies, Inc. (Coralville, IA, USA, sTN29a and sTN29b). Aptamers were internally labeled with Alexa Fluor 647, as previously reported [11]. The sequences of truncated and full-length correspondent aptamers are reported in Supplementary Table S1. Scr, used as a negative control, has the following sequence: 5′UUCGUACCGGGUAGGUUGGCUUGCACAUAGAACGUGUCA3′. 

Before each use, the aptamers dissolved in RNAse-free water at a final concentration of 20 µM were subjected to a heating and cooling step (85 °C for 5 min, on ice for 3 min, 37 °C for 10 min) for ensuring their proper folding.

The secondary structures were predicted by using ViennaRNA Web Services (RNAfold web server, http://rna.tbi.univie.ac.at/cgi-bin/RNAWebSuite/RNAfold.cgi, accessed on 10 January 2022); RNAstructure version 6.2 and the Mfold online web server (http://www.unafold.org/mfold/applications/rna-folding-form.php, accessed on 10 January 2022). 

### 4.3. Cell Binding Analyses

The binding of the short aptamers to TNBC cells was assessed by streptavidin-biotin-based (colorimetric) and flow cytometric assays by using 5′-end biotinylated or fluorescently labeled aptamers, respectively, as previously described [11,35]. The same batch of cells, originally used for the cell-SELEX protocol and for binding analyses with full-length aptamers [11], was used. Briefly, 2.0 × 10^5^ cells, plated in a clear round-bottom 96-well plate (Corning Incorporated, Corning, NY, USA) or maintained in suspension for colorimetric and flow cytometric assay, respectively, were incubated in binding buffer consisting of BlockAid supplemented with yeast tRNA and ultrapure salmon sperm DNA, in the absence (mock-treated) or in the presence of aptamers at the indicated concentrations for 10 min at RT, as reported [11]. The two assays were applied for binding affinity (Kd values) calculation by using the Scr negative control to determine the nonspecific binding. The apparent Kd of aptamer-cell interaction was calculated by fitting the dependence of specific binding on the aptamer concentration to the equation

Y = Bmax × X/(Kd + X), using GraphPad Prism version 6.00. The nonspecific binding value for the Scr was subtracted from every data point.

### 4.4. Confocal Microscopy 

In order to visualize the cell binding/uptake of the aptamers on living cells, cells were seeded on the coverslip (1.0 × 10^5^ cells/well in 24-well) and, after 24 h, were incubated with Alexa Fluor 647-labeled aptamers (500 nM-final concentration) for 10 min at RT. Then, after 3 washes in Dulbecco’s phosphate-buffered saline (DPBS), cells were fixed with 4% paraformaldehyde in DPBS for 20 min, washed 3 times in DPBS and incubated with WGA-Alexa Fluor 488 conjugate (WGA-488, Invitrogen, Carlsbad, CA, USA) as cell membrane staining, for 30 min at RT. Finally, after 3 washes with DPBS, cells were incubated with 1.5 μM 4′,6-Diamidino-2-phenylindole (DAPI, D9542, Sigma-Aldrich, Milan, Italy) and mounted with glycerol/DPBS. 

For internalization experiments, cells were pre-treated with LysoTracker Red DND-99 (1:1000, Invitrogen) in RPMI-1640 medium supplemented with 10% FBS for 1 h at 37 °C and then incubated with Alexa Fluor 647-labeled aptamers for 30 min at 37 °C. After washing, cells were fixed and incubated with WGA-488 and DAPI, as described above. 

Samples were visualized by Zeiss LSM 700 META confocal microscopy equipped with a Plan-Apochromat 63x/1.4 Oil DIC objective. Magnified views were obtained using Image J (v1.46r) software.

### 4.5. Cell Viability

Cells (5.0 × 10^3^ cells/well), previously seeded into a 96-well plate (Corning Incorporated), were treated in the presence of 100 µL cell culture medium containing sTN145, sTN58, sTN29 or Scr (2 µL aptamer, for reaching the final concentration of 400 nM) or 2 µL RNAse-free water (mock-treated). After 24 h incubation, cell viability was assessed by CellTiter 96 AQueous One Solution Cell Proliferation Assay (Promega BioSciences Inc., San Luis Obispo, CA, USA), according to the manufacturer’s instructions.

### 4.6. Aptamer Stability in Human Serum

The stability of sTN145, sTN58 and sTN29 in 80% human serum (Sigma-Aldrich) at 37 °C up to 48 h was assessed as previously reported [11]. To check the RNA integrity, samples were loaded on denaturing polyacrylamide gels (15% polyacrylamide with 7 M urea) that were stained with ethidium bromide and UV-exposed to visualize the RNA bands. Band intensity was quantified using ImageJ (v1.46r) at each time point and expressed as a percentage with respect to time 0 RNA.

### 4.7. Mammosphere Formation Assay

Cells (2 × 10^5^ cells/well into 6-well plates) were treated with sTN145, sTN58, sTN29 or Scr (400 nM-final concentration). After 24 h of treatment, cells were trypsinized, resuspended in Dulbecco’s modified Eagle’s medium/F-12 (DMEM/F-12, Sigma-Aldrich) serum-free medium, and counted. Cells (5 × 10^3^ cells/well) were plated in 24 ultralow attachment plates (Corning Incorporated) and grown in DMEM/F-12 serum-free medium, supplemented with B27 (1:50, Invitrogen), 20 ng/ mL basic fibroblast growth factor and 20 ng/ mL EGF (Sigma-Aldrich). After 10 days, the size and number of primary mammospheres (P0) were analyzed under phase-contrast microscopy (Leica DMI3000 B apparatus). All spheres with a diameter >40 μm were counted and measured in at least 10 fields per condition. Primary mammospheres (P0) were then collected, disaggregated into single-cell suspension mechanically after a short incubation with trypsin (Sigma-Aldrich), and reseeded (5 × 10^3^ cells/well into 24 ultralow attachment plates) in a stem culture medium to form a secondary mammosphere population (P1). Self-renewal activity was evaluated by counting the number of P1 mammosphere/cell-seeded × 100. Cells treated in the same way except without the aptamers (mock-treated) were used as control.

### 4.8. Statistical Analysis

All statistical values were defined using GraphPad Prism version 6.00 by one-way ANOVA followed by Tukey’s multiple comparison test. *p*-value < 0.05 was considered significant for all analyses.

## Data Availability

All data generated or analyzed during this study are included in this article.

## Supplementary Materials

Supplementary materials can be found at https://www.mdpi.com/article/10.3390/ijms23073511/s1.
