# Supplementary material for: Optimization of Short RNA Aptamers for TNBC Cell Targeting
Source: Int J Mol Sci. 2022 Mar 23;23(7):3511. doi: 10.3390/ijms23073511 (PMC8998535; doi:10.3390/ijms23073511)
Supplement: Supplementary file 1 [file ijms-23-03511-s001.zip › ijms-1617778-supplementary.pdf]

Supplementary Table S1. Full-length and short sequence of the three aptamers. The full-length and short sequences of the three TNBC aptamers are reported. Underlined letters indicate the variable region in the aptamer sequence. Bold letters indicate the 2'F-Pys in the aptamer sequence.

**Full-length**		**Short**	
TN58	5'GGGAAGAGAAGGACAUAUGAUGCAACGUUGUGGUCCCGUUUGCACUUUGUUUACGCGCGCAUUGACUAGUACAUGACCACUUGA3'	sTN58	5'GGACAUAUGAUGCAACGUUGUGGUCCCGUUUGCACUUUGUUUACG3'
TN145	5'GGGAAGAGAAGGACAUAUGAUCCUCAGCGCGCAACUUCCCUCCGUUCCCUGCCACGCGUCAUUGACUAGUACAUGACCACUUGA3'	sTN145	5'GGGAAGAGAAGGACAUAUGAUCCUCAGCGCGCAACUUCCCUCCGUUCCC3'
TN29	5'GGGAAGAGAAGGACAUAUGAUCCUGCCCCAACCAUCGCUUCCUCGACGCGCGUUGUCGGCAUUGACUAGUACAUGACCACUUGA3'	sTN29a	5'GGAAGAGAAGGACAUAUGAUCCUGCCCCAACCAUCGCUUCC3'
		sTN29b	5'UGCCCCAACCAUCGCUUCCUCGACGCGCGUUGUCGGCA3'

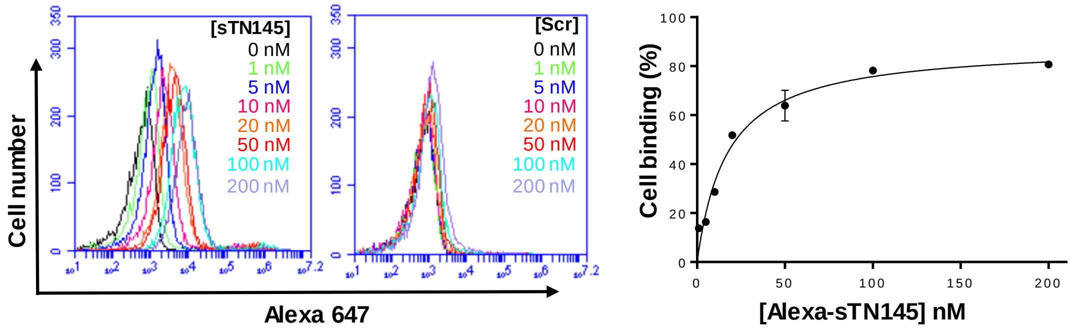

Supplementary Figure S1. Binding analysis of Alexa 647-labeled sTN145 aptamer with MDA-MB-231 cells by flow cytometry. (*Left*) MDA-MB-231 cells were mock-treated or incubated with increasing concentrations of Alexa 647-labeled sTN145 and Scr and binding was analyzed by flow cytometry. (*Right*) The binding curve, which was obtained by plotting the percentage of cells targeted by the Alexa 647-labeled aptamer against varying aptamer concentrations, is shown. Data shown are mean ± SD of three independent experiments.

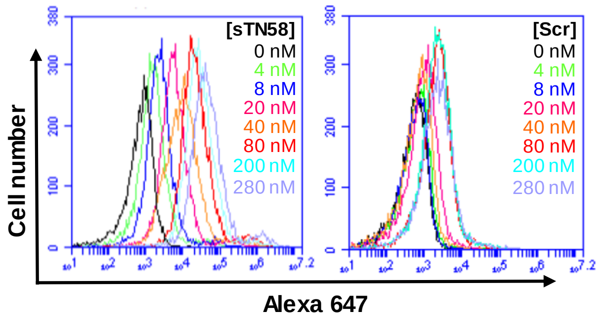

Supplementary Figure S2. Binding analysis of Alexa 647-labeled sTN58 aptamer with MDA-MB-231/cis cells by flow cytometry. Shown is the flow cytometry analysis of increasing concentrations of Alexa 647-labeled sTN58 and Scr incubated with MDA-MB-231/cis cells for Kd calculation (as shown in Figure 3B).

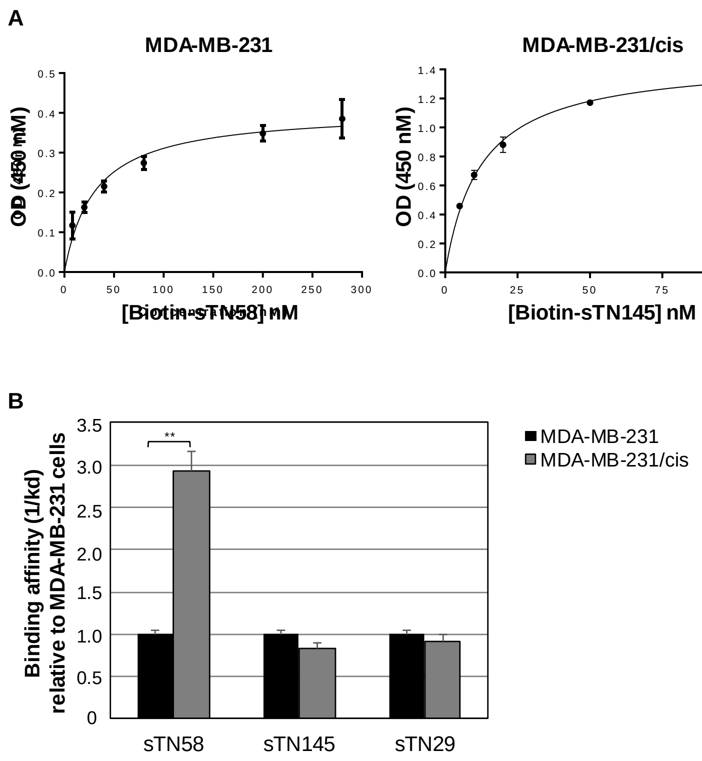

Supplementary Figure S3. Binding affinities of short aptamers vs parental and chemo-resistant MDA-MB-231 cells. (A) Shown is the binding curve of 5'-biotinylated sTN58, sTN145 and sTN29 on indicated cell lines. (B) Binding affinity (1/Kd) of short aptamers to MDA-MB-231/cis cells is expressed relative to the corresponding binding affinity to MDA-MB-231 cells. Note that the Kd values obtained on parental and chemo-resistant cells by using the streptavidin-biotin based assay were taken into account for this analysis. Data shown are mean ± SD of four independent experiments. **p< 0.01.

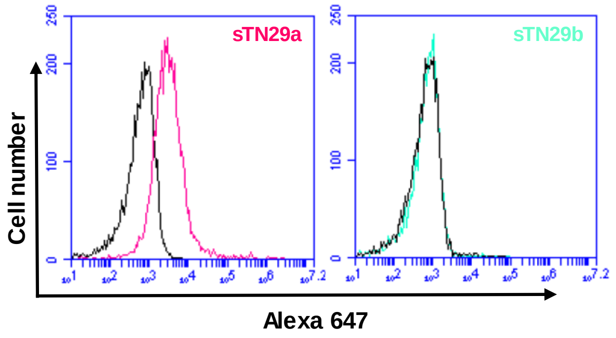

Supplementary Figure S4. Binding analysis of Alexa 647-labeled sTN29a and sTN29b aptamers with MDA-MB-231 cells by flow cytometry. MDA-MB-231 cells were incubated with 20 nM Alexa 647-labeled sTN29a (*left*) or sTN29b (*right*), and binding was analyzed by flow cytometry. The results are shown relative to the control-cells (black line).

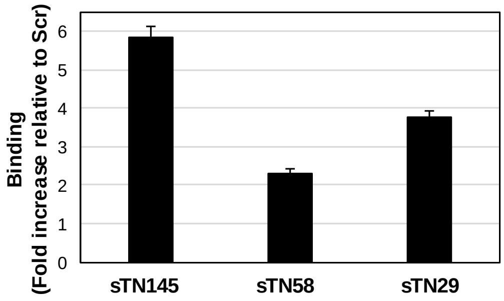

Supplementary Figure S5. Binding of short aptamers to BT-549 cells. Binding of sTN145, sTN58, sTN29 (200-nM final concentration, colorimetric assay) on BT-549 TNBC cells. The results are expressed relative to the background binding detected with the Scr negative control. Bars depict mean ± SD of three independent experiments.
